# Are nurses and patients willing to work with service robots in healthcare? A mixed-methods study

**DOI:** 10.1186/s12912-024-02336-7

**Published:** 2024-10-07

**Authors:** Heba Emad El-Gazar, Shymaa Abdelhafez, Amira Mohammed Ali, Mona Shawer, Talal Ali F. Alharbi, Mohamed Ali Zoromba

**Affiliations:** 1 Nursing Administration Department, Faculty of Nursing, Port-Said University, Port-Said, Egypt; 2 Department of Psychiatric Nursing and Mental Health, Faculty of Nursing, Alexandria University, Alexandria, Egypt; 3 Nursing Education and Advanced Practice Lead, King’s College Hospital London-Jeddah, Jeddah, Saudi Arabia; 4 Technical Institution of Nursing, Mansoura, Egypt; 5 Department of Psychiatric, Mental Health and Community Health, College of Nursing , Qassim University, Buraydah, Saudi Arabia; 6 College of Nursing, Sulaiman Al Rajhi University, Al Bukayriah, Saudi Arabia; 7 College of Nursing, Prince Sattam Bin Abdulaziz University, Al-Kharj, Saudi Arabia

**Keywords:** Healthcare service robots, Mixed-methods study, Nurses’ behavioral intentions to accept robots, Patient willingness to use robots

## Abstract

**Introduction:**

Scholars have become increasingly interested in incorporating robots into healthcare. While there is a growing body of research examining nurses’ and patients’ attitudes towards using robots in healthcare, no prior research has specifically explored their willingness to integrate service robots within the Egyptian healthcare context.

**Aim:**

The aim of this study was twofold: (a) to explore the behavioral intentions of nurses to accept robots in their workplace, and (b) to examine the willingness of patients to use service robots in healthcare settings.

**Methods:**

A mixed-methods study was conducted. Quantitative data were collected from 301 nurses using the Behavioral Intention to Accept Robots in the Workplace Scale and from 467 patients using the Service Robot Integration Willingness Scale through convenience sampling at three tertiary public hospitals in Port Said, Egypt. Qualitative data were obtained through in-depth, semi-structured interviews with 16 nurses, focusing on their perspectives and concerns regarding robot integration. Descriptive analyses were used to analyze quantitative data, and thematic analysis was used to analyze qualitative data.

**Results:**

Quantitative results indicated a moderate level of behavioral intention to use robots among nurses. Patients demonstrated low willingness to use service robots. In the qualitative analysis of the data obtained from the interviews with nurses, three categories (Concerns about Robots, Roles and Competencies, and Potential Benefits) and eight themes (interaction and emotions, maintenance and reliability, job insecurity, role clarity, competence in critical care, trustworthiness, reducing physical strain, and specialized applications) were identified.

**Conclusion:**

The results of this study indicate that nurses’ behavioral intention to accept service robots in healthcare settings is moderate and their acceptance is influenced by various factors related to their concerns about robots, roles and competencies, and potential benefits they could gain. Patients showed a low level of willingness to use service robots in healthcare settings.

**Implication:**

Providing targeted educational programs to nurses and patients, assuring them with the provision of robust maintenance protocols, enhancing their confidence in the capabilities of robots, and defining clear roles for robots are crucial for the successful integration of robots into healthcare settings.

**Supplementary Information:**

The online version contains supplementary material available at 10.1186/s12912-024-02336-7.

## Introduction

The integration of artificial intelligence (AI) into healthcare settings is progressively expanding worldwide. AI is playing an increasingly pivotal role in transforming health service deliveries, enhancing quality, efficiency, and setting new expectations [1]. Global expenditures on these technologies in healthcare are projected to exceed $36 billion by the year 2025, and they are expected to be available to everyone, everywhere [2]. Within the realm of AI, the use of service robots—software-programmed machines capable of sensing and interacting with their environment to perform a variety of tasks—marks a significant shift in healthcare delivery [3]. Robots can perform tasks autonomously based on their programming, operating independently without human intervention [4]. There are various applications for robots in healthcare, from participating in exercise programs to potentially providing companionship and emotional support [5].

Recently, the integration of robots in nursing practice has garnered increasing scholarly interest [1, 4]. Previous research has demonstrated that robots can assist nurses in delivering efficient patient care, such as handling supplies, materials, and medications [6], and monitoring patient mobility and activities [7]. Additionally, robots could help in job crafting by reducing workload and maximizing job resources [8], which in turn increases passion for work [9]. This support could improve nurses’ health and job satisfaction, thereby potentially reducing turnover rates [3].

The integration of robots into healthcare settings is not without its controversies [10]. Despite recognition in the literature for their precision and efficiency, robots in nursing practice face certain limitations [3]. For example Stokes and Palmer have raised ethical concerns regarding the use of robots in healthcare [11], while Tu et al. have highlighted potential issues such as loss of autonomy and negative psychological outcomes [12]. These issues have prompted scholars to question whether nurses and patients would feel comfortable entrusting their well-being to robots [13]. Given these concerns, it is crucial to investigate nurses’ and patients’ intentions and apprehensions about working with robots. Therefore, this study aims to (a) explore the behavioral intentions of nurses to accept robots in their workplace, and (b) examine the willingness of patients to use service robots in healthcare settings.

This study contributes to the nursing literature by identifying the actual level of acceptance among nurses and patients toward robot integration in nursing practice. Additionally, it pinpoints the concerns nurses have regarding robot integration, which may hinder nurse-robot cooperation. By effectively addressing these concerns, this research responds to scholarly calls for exploring barriers to the acceptance of robots [14]. As a result, nurses and patients might become more welcoming of the integration of robots in nursing practice.

## Theoretical background and prior research

Robotic technology is rapidly expanding across healthcare environments, propelled by innovations and the pressing requirement for enhanced healthcare efficiency [15]. Robots, defined as programmable machines capable of performing complex actions autonomously or semi-autonomously, are now common in various medical applications, from surgery to routine care [16]. In nursing, the transformative potential of robots in care practices has attracted significant scholarly and practical attention. Empirical research has identified several ways in which robots can enhance nursing practices. For example, prior studies have demonstrated that robots assist nurses by delivering medication, monitoring patients, and providing routine nursing treatments, which can extend and enrich the scope of nursing work [4]. Additionally, robots are recognized for their effectiveness in minimizing non-value-added activities [7]. Another line of research has studied the impact of robots in anticipation of the empathic behavior presented by artificial humanoid robots [13] and their ethics in robot caring [11].

Despite these valuable insights, a critical question remains regarding the attitudes and acceptance levels of nurses toward the integration of AI and robotics in healthcare. Most of the existing research has focused on nursing students [17], which may not fully represent the views of practicing nurses and patients who directly interact with this technology daily. An exception is a cross-sectional study that measured nurse managers’ opinions on AI and robot nurses, identifying literacy as a major concern [2]. However, comprehensive research that measures actual acceptance levels and practical integration experiences among frontline nurses and patients remains sparse.

Given that nurses are primary care providers and patients are the recipients of such technology, it is crucial to assess their actual acceptance levels. This gap underscores the need for empirical research that captures real-world experiences and responses to robotic technologies in healthcare settings. Hence, this study aims to bridge this gap by empirically examining the behavioral intentions of nurses and the willingness of patients to integrate service robots within the Egyptian healthcare context. By doing so, we respond to scholarly calls for more empirical research to explore the acceptability and effective implementation of AI technologies [2].

Moreover, this study includes a quantitative analysis that measures the perspectives and concerns of nurses regarding the integration of robotic technologies into healthcare settings. This approach allows for a comprehensive understanding of the factors influencing acceptance and identifies potential barriers to successful integration. By addressing these specific concerns, the study aims to facilitate smoother adoption of robots in healthcare, ensuring enhanced provider satisfaction and improved patient care quality.

Additionally, this research specifically explored whether nurses’ and patients’ willingness to use service robots in healthcare settings varied based on their demographic characteristics. In the field of healthcare technology acceptance, demographic variables play a pivotal role in shaping nurses’ attitudes and behavioral intentions [18]. For instance, age and gender differences in the acceptance of artificial technology among nurses have been well-documented, with studies indicating that younger female nurses are more likely to accept AI technologies [18, 19]. Similarly, educational background has been linked to varying attitudes towards AI among nurses [20, 21]. Nurse acceptance of the use of AI also varies according to their nursing experience [22] and the department in which they work within the hospital [23]. Moreover, a study by Liu et al. revealed that individual characteristics significantly influenced patients’ continuance intention to use AI-powered service robots in hospitals [10]. Furthermore, patients’ attitudes toward the use of AI have been shown to vary according to their marital status [24]. These findings underscore the importance of considering demographic variables when investigating technology acceptance, such as robots in healthcare.

## The study

### Aim

The aim of this study was twofold: (a) to explore the behavioral intentions of nurses to accept robots in their workplace, and (b) to examine the willingness of patients to use service robots in healthcare settings.

### Study questions

This study seeks to answer the following questions:

- What is the level of behavioral intention among nurses to accept robots in their workplace?
- What are the perspectives and concerns of nurses regarding the integration of robots into healthcare settings?
- What is the level of willingness among patients to use service robots in healthcare settings?
- Do nurses’ and patients’ willingness to use service robots in healthcare settings differ based on their demographic characteristics?

## Methods

### Study design

This study utilizes a mixed-methods approach with a convergent parallel design to obtain diverse yet complementary data, facilitating a comprehensive understanding of the research problem. In this design framework, qualitative and quantitative data are collected simultaneously but independently, ensuring equal emphasis on both types of data. Each dataset is analyzed separately and subsequently integrated during the evaluation phase to enhance the depth and breadth of the findings [25]. Throughout the research, adherence to the STROBE checklist for cross-sectional studies and the COREQ guidelines for qualitative research was rigorously maintained.

### Participants and setting

The quantitative part of this research aimed to assess the readiness of nurses and patients for the integration of robots in healthcare settings. Data were collected using a convenience sampling method from clinical nurses and patients at three tertiary public hospitals in Port Said, Egypt. To determine the required sample size for nurses, we used Yamane’s (1967) formula for finite population [26]:

$$\:{\rm{n}} = \frac{{\rm{N}}}{{1 + N{{\left( e \right)}^2}}}\: = \:\frac{{1219}}{{1 + 1219{{(0.05)}^2}}} = {\rm{301\ nurses}}$$

Here, “n” represents the necessary sample size, “N” is the total population size (*N* = 1219), and “e” denotes the error term set at 0.05, yielding a minimum required sample of 301 nurses.

For patients, the appropriate sample size was calculated using Cochran’s formula for infinite population [27]:

$${\rm{n}} \ge \:\frac{{{z^2}\: \times \:\sigma {\:^2}}}{{{d^2}}} = \:\frac{{{{1.96}^2}\: \times \:0.68{\:^2}}}{{{{0.062}^2}}} = {\rm{462}}\,{\rm{patients}}$$

Here, “n” indicates the required sample size, “z” is the standardized normal deviation corresponding to a 95% confidence level and a 5% alpha level (z = 1.96, two-tailed), “σ” is the expected standard deviation in the population (0.68 from the pilot study), and “d” is the acceptable margin of error for the mean (d = 0.062, based on the pilot study mean of 2.08 and a 0.03 margin of error). To account for potential dropout, the sample sizes were increased by 20%, resulting in 361 surveys distributed to nurses and 554 surveys to patients. Of the distributed surveys, 306 from nurses and 467 from patients were validly returned.

We applied Yamane’s formula for the nurse population, as the number of the target population is known and finite. Meanwhile, we used Cochran’s formula for the patient population, which is considered potentially infinite. This approach ensures that the sampling methodology is both robust and appropriate for the diverse conditions of the nurse and patient populations under study.

For the qualitative study, 16 nurses were recruited from two different tertiary public hospitals in Port Said, Egypt. Sample size saturation was achieved when no new information was gleaned from subsequent interviews [28]. Inclusion criteria for nurses were licensed staff nurses who were on duty during the study period and had at least one year of tenure in the nursing profession. Exclusion criteria were nurses or patients who declined to participate in the study.

### Study Instrument – quantitative data

Quantitative data were collected using two distinct scales. The Behavioral Intention to Accept Robots in the Workplace Scale (BIARW) was administered to nurses, and the Service Robot Integration Willingness Scale (SRIW) was used for patients. Both surveys were translated into Arabic through a back-translation procedure [29]. Participants involved in the pilot testing were excluded from the main sample. All items on both scales were rated on a five-point Likert scale, ranging from 1 (Strongly Disagree) to 5 (Strongly Agree).

#### BIARW

Developed by Sinha et al., the BIARW is a three-item scale used to assess nurses’ intentions to accept robots in their workplace [30]. Example items include, “I am willing to accept robots in my workplace” and “I am likely to interact with robotics in my workplace.”

#### SRIW

Developed by Lu et al., the SRIW used to measures patients’ willingness to use service robots in healthcare settings [31]. This scale comprises 36 items distributed across six dimensions: (a) performance efficacy (7 items), (b) intrinsic motivation (6 items), (c) anthropomorphism (7 items), (d) social influence (7 items), (e) facilitating conditions (4 items), and (f) emotions (5 items). Example items include, “Information provided by robots is more accurate with less human errors in healthcare services” and “If I use robots in healthcare settings, I will feel satisfied.”

### Study instrument – qualitative data

For the qualitative part, semi-structured interview forms consisting of eight open-ended questions, developed by the researchers specifically for this study (Supplementary file 1), were employed to explore nurses’ perspectives and concerns regarding the integration of robots into healthcare settings.

### Validity and reliability

Face and content validity of the translated scales used for quantitative data collection were assessed by involving seven experts and professors in nursing. These experts were asked to evaluate whether the scales appeared to measure the intended constructs, thus ensuring face validity. Content validity was assessed by having the experts rate each item on a 4-point Likert scale, ranging from 1 (not relevant) to 4 (highly relevant). If the item-level Content Validity Index (I-CVI) was ≥ 0.78 and the scale-level CVI average (S-CVI/Ave) was ≥ 0.90, the content validity of the scale was considered satisfactory [32]. The expert panel confirmed the face and content validity, with I-CVI scores ranging from 0.94 to 1.00 for the BIARW and 0.93 to 1.00 for the SRIW. The S-CVI/Ave was 0.97 for the BIARW and 0.95 for the SRIW, demonstrating acceptable content validity.

The reliability of the scales was determined using Cronbach’s alpha coefficient, which was 0.853 for the BIARW and 0.966 for the SRIW, indicating acceptable internal consistency [33].

### Data collection

The study was conducted between January and May 2024.

### Quantitative data

Quantitative data were collected by three trained research assistants after obtaining permission from hospital administrators. Eligible participants, including nurses and patients, were personally approached and given a thorough orientation about the study’s aims, potential risks, and benefits. Only those who consented to participate and signed informed consent forms were provided with a closed package containing the survey. The questionnaire was administered using a paper-and-pencil format. The surveys were collected immediately upon completion.

### Qualitative data

Qualitative data were collected by researchers experienced and trained in qualitative research. Sixteen in-depth, semi-structured interviews were conducted online with nurses. A semi-structured format was chosen to focus on specific dimensions while allowing respondents to introduce new insights related to the topic. Prior to the interviews, written informed consent was obtained from eligible nurses. All interviews were audio-recorded and later transcribed by two different researchers upon completion. On average, each interview lasted 60 min. Data collection continued until data saturation was achieved.

### Statistical analysis

Qualitative data were analyzed using thematic analysis [34] to explore and categorize nurses’ perspectives and concerns regarding the integration of robots in healthcare settings. Two researchers independently reviewed the transcripts to identify significant words and phrases, which were then coded to generate themes and sub-themes. They met to discuss their initial findings and themes, refining and adjusting them as necessary until reaching a consensus. This iterative process ensured the themes accurately reflected the data. The discussion and analysis process spanned three meetings, each averaging three hours.

Quantitative analysis was performed using SPSS 27.0 software, with a *p*-value of < 0.05 considered statistically significant. Descriptive statistics were used to outline participant characteristics and assess the levels of nurses’ behavioral intentions toward accepting robots in their workplace, as well as patients’ willingness to use service robots. Independent samples t-tests and one-way ANOVA were utilized to compare nurses’ behavioral intentions and patients’ willingness based on demographic characteristics. When ANOVA indicated statistical significance, subsequent pairwise comparisons were conducted using Tukey’s HSD post-hoc tests.

### Rigor and reflexivity of qualitative analysis

Participation in this research was entirely voluntary. To ensure conformability, a single researcher conducted all the interviews. After each interview, participants were invited to add new information or clarify their responses. To ensure the dependability and validity of the data, two researchers independently identified the main themes and sub-themes, which were then discussed until consensus was achieved. Sample quotes were directly extracted from the interview reports.

## Results

### Quantitative results

#### Results from nurses

As shown in Table 1, the majority of participants were female (*n* = 218, 71.2%), married (*n* = 200, 65.4%), and held an associate-level education (*n* = 153, 50.0%). Among the 306 participants, 43.8% (*n* = 134) worked in medical or surgical units. Most participants were aged between 30 and 40 years (*n* = 116, 37.9%) and had less than 10 years of professional experience (*n* = 166, 54.3%). The mean score for behavioral intention to accept robots among the participating nurses was 2.65 (SD = 0.89) out of 5, indicating a moderate level of intention.

One-way ANOVA with post hoc comparisons revealed statistically significant differences in nurses’ behavioral intention to accept robots based on marital status (F(3, 302) = 3.37, *p* = 0.019, $\:{{\upeta\:}}_{p}^{2}$ = 0.032; mild effect size) and education level (F(2, 303) = 9.94, *p* < 0.001, $\:{{\upeta\:}}_{p}^{2}$ = 0.062; moderate effect size). Specifically, single and widowed nurses demonstrated a significantly higher behavioral intention to accept robots compared to married and divorced nurses. Additionally, nurses with an associate degree or higher showed a significantly greater intention to accept robots than those with a diploma. The t-test further indicated that male nurses had a significantly higher behavioral intention to accept robots compared to female nurses (t(304) = 2.68, *p* = 0.008, d = 0.33; mild effect size).

**Table 1:** Characteristics of participating nurses and their behavioral intention to accept robots in the workplace (*N* = 306)

Characteristic	Category	no	%	Behavioral intention to accept robots	
				M (SD)	t/F (P)
**Age (years)**	< 30	107	35	2.78 (0.93)	F = 2.24 (0.108)
	30–40	116	37.9	2.63 (0.84)	
	> 40	83	27.1	2.51 (0.89)	
**Gender**	Male	88	28.8	2.86 (0.84)	t = 2.68 (0.008)
	Female	218	71.2	2.57 (0.90)	
**Marital status**	Single^a^	85	27.8	2.82 (1.02)	F = 3.37 (0.019) a, d > b, c
	Married^b^	200	65.4	2.59 (0.85)	
	Divorced^c^	18	5.9	2.43 (0.48)	
	Widowed^d^	3	0.9	3.78 (0.69)	
**Education**	Diploma^a^	76	24.8	2.27 (0.67)	F = 9.94 (< 0.001) b, c > a
	Associate^b^	153	50.0	2.75 (0.94)	
	Bachelor or above^c^	77	25.2	2.83 (0.87)	
**Unit**	Intensive care	64	20.9	2.51 (0.81)	F = 1.46 (0.227)
	Emergency	68	22.2	2.78 (0.81)	
	Medical or surgical	134	43.8	2.69 (0.96)	
	Other	40	13.1	2.53 (0.89)	
**Nursing tenure (years)**	< 10	166	54.3	2.70 (0.91)	F = 0.59 (0.556)
	10–20	53	17.3	2.58 (0.89)	
	> 20	87	28.4	2.60 (0.86)	
Behavioral intention to accept robots in the workplace				2.65 (0.89)	

F = one-way analysis of variance; t = independent sample t-test

^abcd^ Differences between the means by Turkeys’ HSD post hoc test

#### Results from patients

As shown in Table 2, most participating patients were male (*n* = 274, 58.7%) and aged between 35 and 44 years (*n* = 175, 37.5%). Among the 467 participants, 61.9% were married (*n* = 289), and 44.1% held an associate degree (*n* = 206). The majority of them (*n* = 374, 80.1%) were suffering from acute health conditions. None of the participants had previous experience with robots. One-way ANOVA with post hoc comparisons revealed statistically significant differences in patients’ willingness to use robots based on marital status (F(3, 463) = 4.25, *p* = 0.006, $\:{{\upeta\:}}_{p}^{2}$ = 0.027; mild effect size), with single patients showing greater willingness than their married counterparts.

**Table 2:** Characteristics of participating patients and their willingness to use service robots (*N* = 467)

Characteristic	Category	no	%	Behavioral intention to accept robots	
				M (SD)	t/F (P)
**Age (years)**	< 25	40	8.6	2.20 (0.71)	F = 2.25 (0.062)
	25–34	125	26.8	2.32 (0.97)	
	35–44	175	37.5	2.10 (0.71)	
	45–55	102	21.8	2.08 (0.71)	
	> 55	25	5.4	1.99 (0.44)	
**Gender**	Male	274	58.7	2.17 (0.86)	t = 0.54 (0.587)
	Female	193	41.3	2.13 (0.66)	
**Marital status**	Single^a^	128	27.4	2.37 (0.99)	F = 4.25 (0.006) a > b
	Married^b^	289	61.9	2.08 (0.68)	
	Divorced^c^	35	7.5	2.10 (0.66)	
	Widowed^d^	15	3.2	2.09 (0.68)	
**Education**	High school	115	24.6	2.11 (0.66)	F = 2.44 (0.064)
	Associate	206	44.1	2.26 (0.88)	
	Bachelor	128	27.4	2.05 (0.74)	
	Postgraduate	18	3.9	2.02 (0.42)	
**Health Condition**	Acute	374	80.1	2.18 (0.83)	t = 1.03 (0.304)
	Chronic	93	19.9	2.08 (0.57)	

Note: All participants had no previous experience with robots.

F = one-way analysis of variance; t = independent sample t-test

^abcd^ Differences between the means by Turkeys’ HSD post hoc test

Table 3 indicates the mean score of patients’ willingness to use service robots in healthcare settings. The total mean score was 2.16 (SD = 0.78) on a scale of 1–5, which indicates a low willingness to use service robots. Among the dimensions evaluated, emotions scored the highest with a mean of 2.80 (SD = 0.83), followed by social influence at 2.09 (SD = 0.93), while facilitating conditions scored the lowest with a mean of 2.01 (SD = 0.89).

**Table 3:** Patients’ willingness to use service robots (*N* = 467)

Scale	M (SD)
Willingness to use service robots total score	2.16 (0.78)
Performance efficacy	2.02 (0.89)
Intrinsic motivation	2.08 (0.96)
Anthropomorphism	2.05 (0.94)
Social influence	2.09 (0.93)
Facilitating conditions	2.01 (0.89)
Emotions	2.80 (0.83)

### Qualitative data

The mean age of the nurses who participated in the qualitative interviews was 34.81 years (SD = 9.21). Of the participants, 75.0% were female (*n* = 12), 43.8% held a diploma (*n* = 7), and 31.3% held a bachelor’s degree or higher (*n* = 5). Additionally, 68.8% were married (*n* = 11), and the average years of professional experience was 15.38 ± 8.72 years.

#### Categories, themes, and subthemes

As a result of the qualitative analysis of the data obtained from the interviews with nurses, three categories (Concerns about Robots, Roles and Competencies, and Potential Benefits), eight themes, and thirteen subthemes were identified (Table 4).

### Category 1: concerns about Robots

#### Theme 1: Interaction and Emotions

Nurses (n: 12) mentioned concerns about the interaction and emotional aspects of using robots in patient care. They reported that robots are unable to provide the empathetic and compassionate care that human nurses offer, which is crucial for building trust and rapport with patients. Additionally, robots may strictly follow predefined steps without flexibility, leading to concerns about their ability to address individual patient needs effectively. This standardized approach to care may not cater to the personalized needs of each patient. Furthermore, as systems that cannot be negotiated with, robots may struggle in situations requiring human judgment and adaptability.

……the robot has no ability to interact. It’s a machine that takes inputs and gives results, so it will execute the care plan without adapting to the specific needs of each patient. Patients are not all the same. The robot lacks flexibility; it will always follow predefined steps. (N8. F)

#### Theme 2: Maintenance and Reliability

Nurses (n: 7) stated concerns regarding the maintenance and reliability of robots in patient care settings. They emphasized the potential for robots to malfunction and require maintenance, which could disrupt the continuity of care. The need for regular maintenance and the possibility of downtime were highlighted as significant issues. Additionally, there were concerns about patients inadvertently damaging robots, raising questions about their durability and the impact on patient safety and care delivery.

…. if the robot malfunctions or needs maintenance during a critical operation, it could cause delays and complications. I also worry about patients accidentally damaging the robot, which would not only be costly but also affect the care we can provide. (N14. F)

#### Theme 3: Job Security

Nurses (n: 9) expressed concerns about the impact of robots on job security in the nursing profession. They feared that the integration of robots could lead to the replacement of human nurses, reducing job opportunities and altering the traditional roles and responsibilities of nursing staff. This apprehension was tied to the broader uncertainty about how the roles of robots and human nurses would be defined and managed in the healthcare setting.

…. I am worried that robots might take over our jobs. It’s not just about the tasks they can perform, but also about how our roles will change. Will there still be a place for us, or will we be sidelined. (N6. M)

### Category 2: concerns about Robots

#### Theme 4: Role clarity

Nurses (n: 11) expressed uncertainty about how the roles of robots and human nurses will be defined and managed. They were concerned about the division of responsibilities and accountability for errors. It is unclear who would be held responsible for errors caused by robots – whether it would be the nurse overseeing the robot.

*“……there is a lot of uncertainty about how the roles will be divided between us and the robots. Who will be accountable if something goes wrong? (N4. F)”*.

#### Theme 5: Competence in Critical Care

Nurses (n: 14) stated they had doubts about whether robots have the necessary skills to manage critical patients and situations effectively. They stated they did not think the robot’s ability to handle complex and unpredictable scenarios could match the human expertise and quick decision-making required in such cases.

……I do not think that the robot’s competence in critical care situations is adequate. Handling critical patients requires nuanced understanding and adaptability, which I don’t believe a robot can provide. In emergencies, quick, informed decisions are crucial, and I’m not confident a robot can meet these demands. (N12. M)

#### Theme 6: Trustworthiness

Nurses (n: 10) reported skepticism about robots’ ability to make nuanced decisions, especially in critical care scenarios. They are concerned that robots may lack the capacity to exercise the judgment and discernment necessary for complex decision-making in high-stakes situations.

…. robots might be efficient in some tasks, but when it comes to critical decision-making, especially in emergency situations, I am skeptical. The subtlety and depth of understanding required to make the right call in such scenarios is something I believe only a human can provide. (N16. F)

### Category 3: potential benefits

#### Theme 7: Reducing Physical Strain

Nurses (n: 6) stated that robots could alleviate physical strain by performing repetitive or physically demanding tasks, which could help reduce the risk of injury and fatigue among nursing staff.

…. Robots could be very helpful in taking over some of the more physically demanding tasks, like lifting patients or handling heavy equipment. This would reduce the physical strain on us and help prevent injuries. (N9. F)

#### Theme 8: Specialized Applications

Nurses (n: 8) stated the potential for robots to be particularly beneficial in specialized applications, such as minimizing staff exposure to radiation and enhancing safety in radiology departments.

*….* In departments like radiology, where there’s a risk of radiation exposure, robots could be invaluable. They can handle tasks that would otherwise put us at risk, improving overall safety. *(N5. F)*

**Table 4:** Categories, themes, and subthemes related to nurses’ acceptance of robots in workplace

Category	Theme	Subtheme	Details
Concerns about Robots	Interaction and Emotions	Lack of human emotions and interaction	Robots lack the ability to interact dynamically with patients.
		Lack of Empathy	Robots cannot provide the empathetic and compassionate care that human nurses offer.
		Lack of resilience	Robots may strictly follow predefined steps without flexibility, which could be problematic for individual patient needs.
		Lack of Negotiation Capability	Robots are systems that cannot be negotiated with, which could be problematic in situations requiring human judgment.
	Maintenance and reliability	Downtime	Potential for robots to malfunction, require maintenance and disrupting care.
		Misuse	Concerns that patients might inadvertently damage robots, raising questions about their durability.
		Reliability and Safety	Concerns about robots failing during critical operations, which lead to delays or complications.
		Extensive training required	Extensive training required for nursing staff to operate robots effectively.
	Job insecurity	Job replacement	Fears that robots replace human nurses in their job.
Roles and competencies	Role clarity	Role of Robots vs. Human Nurses	There is uncertainty about how the roles of robots and human nurses will be defined and managed.
		Accountability for Errors	It is unclear who is responsible for errors caused by robots, whether the nurse or the robot itself.
	Competence in Critical Care	Competence in dealing with critical patients	Doubts if robots have the necessary skills to manage critical patients and situations effectively.
	Trustworthiness	Decision-making inability	Skepticism about robots’ ability to make nuanced decisions, especially in critical care scenarios.
Potential Benefits	Reducing Physical Strain		Robots could alleviate physical strain by performing repetitive or physically demanding tasks.
	Specialized Applications		Robots could minimize staff exposure to radiation and enhance safety in radiology departments.

## Discussion

The acceptance of service robots in healthcare settings reveals complex, multifaceted perspectives shaped by practical, emotional, and professional considerations [35]. This study aimed to explore the behavioral intentions of nurses to accept robots in their workplace and to examine the willingness of patients to use service robots in healthcare settings.

The results indicate a moderate level of behavioral intention among nurses to accept robots in their workplace. This may be due to the integration of new aspects, such as robots, which can encounter resistance to change [36]. These findings align with previous studies that have shown a moderate readiness among nurses to embrace AI applications in nursing [37, 38]. Interestingly, the adoption of robot nurses can be further encouraged [39]. Therefore, providing in-service training and increasing awareness about the benefits and usage of robot nurses may help healthcare organizations enhance nurses’ acceptance of service robots in healthcare settings.

Furthermore, findings from the quantitative data showed that marital status influences nurses’ willingness to accept robots in the workplace. Specifically, single and widowed nurses exhibit a significantly higher intention to accept robots compared to their married and divorced counterparts. One possible explanation for this finding could be that single and widowed nurses may have fewer family responsibilities, potentially making them more open to new technologies and workplace changes. Meanwhile, married and divorced nurses might be more cautious or resistant to change due to their existing commitments and the stability they seek in both their personal and professional lives. However, this contrasts with previous findings that showed marital status did not influence nurses’ attitudes toward AI in nursing practice [19].

Additionally, the study found that male nurses with an associate degree or higher exhibited a significantly greater intention to accept robots than female nurses with a diploma. This result is consistent with research by Alruwaili et al. which highlighted variations in attitudes toward AI among nurses based on their education level [19]. Furthermore, another study showed that nurses with postgraduate education had a more positive attitude toward using AI in nursing practice [20].

Interviews with nurses support these findings, showing that most nurses have concerns about robots’ ability to interact with and display emotions toward patients. This aligns with a prior study that found intelligent humanoid robots capable of displaying empathy, interacting, and responding in a humanlike manner are essential for integrating robots into nursing practice [13]. Additionally, it aligns with the findings of Ergin et al., who reported that nurse managers also believe robots cannot meet the social and emotional needs of patients [2].

Maintenance and reliability were additional concerns among nurses. They highlighted the potential for robots to malfunction and require regular maintenance, which could disrupt the continuity of care. Developing robust maintenance protocols and providing adequate training for staff on troubleshooting and maintaining these machines can help mitigate these issues. This aligns with findings from Kato et al., who revealed that the maintenance of robots is one of the challenges for introducing robotic care equipment [40]. Nurses also have concerns about job security, fearing that robots might replace human roles. This view contrasts with that of nurse managers, who indicated that robot nurses would not replace human nurses [2].

Additionally, the specific roles and functions of robots are also a point of confusion among nurses. They expressed concerns about how tasks would be divided between nurses and robots. This concern echoes prior nursing literature, which highlighted the importance of clearly identifying the division of tasks between nurses and robots [11]. Nurses also expressed doubts about the competence of robots in critical care scenarios, reporting that they find it difficult to trust the system, especially in emergency situations. These results align with previous studies showing that trust in AI plays a crucial role in the intention to use it in healthcare [10, 37]. In non-nursing contexts, research has also shown that one pitfall to accepting robots in the workplace is employees’ inability to trust them [41].

Despite the concerns, several potential benefits of integrating robots in healthcare were identified. Nurses acknowledged that robots could significantly reduce physical strain by taking over repetitive and physically demanding tasks. This could help prevent injuries and reduce fatigue among nursing staff, thereby enhancing their overall efficiency and well-being. Moreover, they stated that robots could be particularly beneficial in specialized applications, such as minimizing staff exposure to radiation in radiology departments and enhancing safety in other high-risk areas. By handling tasks that pose health risks to humans, robots can create a safer working environment for healthcare professionals. This is consistent with an integrative review that indicated robots and automated devices can play a role in alleviating the workload of nurses, thereby facilitating their use [4]. Additionally, a study by Chang et al. showed that nurses perceive that robots enable them to focus more on professional task engagement [3].

Regarding patient willingness to use service robots, the study showed a low willingness among patients to use service robots. This is an important finding, as patients are the recipients of these services. If patients are unwilling or unable to work with robots, this could undermine the effectiveness and implementation of robot services in healthcare settings. This aligns with the results of Laakasuo et al., who found that patients were more accepting of human nurses compared to robot nurses [1]. Additionally, previous studies have shown that patients’ intentions to use robots in hospitals depend on various factors, including ease of use, independent personality, and trust in AI techniques [10].

The study results also showed a variation in patients’ willingness to use service robots based on their marital status, with single patients demonstrating greater willingness than their married counterparts. This may be because single patients feel more comfortable or open to interacting with new technologies, such as service robots, as they might have fewer immediate concerns about how these technologies could impact family dynamics or caregiving responsibilities. Conversely, married patients might be more cautious or skeptical about relying on robots for care, possibly due to concerns about the quality of care or the potential impact on their family life. Married patients may prioritize human interaction in healthcare settings, which they perceive as more reliable or emotionally supportive. In line with these findings, a study by Liu et al. asserted that personal individual characteristics influence patients’ continuous intention to use service robots in healthcare [10].

### Practical implications

This study reveals numerous practical implications for healthcare organizations seeking to implement service robots into their work processes. Our findings indicate that nurses have a moderate level of behavioral intention to accept robots in their workplace. To increase their willingness, healthcare administrators should provide in-service training on AI applications, including robots, to enhance nurses’ literacy in AI, thereby fostering greater acceptance of service robots in healthcare settings.

Additionally, nurses reported concerns that affect their willingness to work with robots. One major concern among nurses is about robots’ ability to interact with and display emotions toward patients. It is important to inform nurses about intelligent humanoid robots [13], which can effectively communicate, interact, and respond empathetically and flexibly to patient needs. Educating nurses about these advanced capabilities can help alleviate concerns about the lack of human-like interaction and emotional display.

Another finding related to robot maintenance indicates that robust maintenance protocols could enhance nurses’ willingness to work with robots. Ensuring that nurses are aware of and confident in these protocols can mitigate fears about potential malfunctions and the disruption of care continuity. Nurses also expressed concerns about job security with the presence of robots. Assuring nurses that robots are tools designed to augment human capabilities rather than replace them can help alleviate fears about job displacement. Furthermore, there are concerns about the roles and competencies of robots. It is important to provide clear guidelines about the specific roles and functions of robots in healthcare settings. Identifying the tasks that robots can perform and those that require human supervision can reduce confusion and enhance acceptance among nurses.

Regarding patients, the study showed a low willingness to use service robots. To address this, integrating robots in a pilot phase and ensuring they are user-friendly could be helpful. Providing awareness sessions for patients about the benefits of robots and how to interact with them can enhance their willingness to use these technologies. Educating patients about the ease of use and the potential improvements in care quality, well as building trust in AI technologies can foster a more favorable perception of service robots.

### Limitations

The findings of this study are restricted to the views and experiences of nurses and patients who participated in the research. The study, conducted with nurses and patients, was carried out in public tertiary hospitals in Port Said, Egypt, using convenience sampling. Thus, it is difficult to generalize the findings to other regions or types of healthcare facilities, such as private hospitals or clinics. Future studies should include a more diverse range of healthcare settings and employ random sampling methods to enhance the generalizability of the results. Additionally, expanding the research to include different cultural contexts and a broader geographic scope would provide a more comprehensive understanding of the factors influencing the acceptance and integration of service robots in healthcare.

## Conclusions

This study aimed to explore the behavioral intentions of nurses to accept robots in their workplace and to examine the willingness of patients to use service robots in healthcare settings. The results of this study indicate that nurses’ behavioral intention to accept service robots in healthcare settings is moderate. Nurses’ acceptance is influenced by various factors, including concerns about interaction and emotions, maintenance and reliability, job security, role clarity, competence in critical care scenarios, and trustworthiness. Additionally, nurses acknowledge potential benefits of robots such as reducing physical strain and specialized applications. Patients showed a low level of willingness to use service robots in healthcare settings. These findings highlight the necessity for targeted educational programs to enhance AI literacy among nurses and patients, robust maintenance protocols, and clear communication about the roles and capabilities of service robots to foster their acceptance and integration in healthcare. Future research should focus on longitudinal studies to assess changes in perceptions among nurses and patients over time, especially following direct exposure to service robots. Additionally, further studies in diverse cultural and healthcare contexts are essential to provide a more comprehensive understanding of the global readiness for healthcare service robots.

## Electronic supplementary material

Below is the link to the electronic supplementary material.

Supplementary Material 1

## Data Availability

Upon request for scientific purposes, the researcher of correspondence will provide researchable information of the research.
