# Supplementary material for: Are nurses and patients willing to work with service robots in healthcare? A mixed-methods study
Source: BMC Nurs. 2024 Oct 7;23:718. doi: 10.1186/s12912-024-02336-7 (PMC11460052; doi:10.1186/s12912-024-02336-7)
Supplement: Supplementary file 1 — Supplementary Material 1 [file 12912_2024_2336_MOESM1_ESM.docx]

Supplementary file 1. Semi-structured in-depth individual interview questions.

**Table 1. Semi-structured In-depth Individual Interview Questions**

| **Category** | **Questions** |
| --- | --- |
| **Introduction** | What comes to mind when you think about the use of robots in healthcare? |
| **Transition** | How did you initially feel about the idea of robots being integrated into your workplace? |
| **Main (Key)** | What are your main concerns regarding the integration of robots into healthcare settings? |
|  | Can you describe any specific instances where you felt either positive or negative about using robots in your professional practice? |
|  | How do you think the use of robots might impact your daily tasks and responsibilities as a nurse? |
|  | What potential benefits do you see in integrating robots into healthcare? |
|  | What are your thoughts on the training and support provided by your organization for working with robots? |
|  | If there were improvements to be made in your training for working with robots, what would they be? |
|  | How do you think the introduction of robots will affect the patient-nurse relationship? |
| **Fixed** | Can you elaborate on that? |
|  | Can you give a specific example? |
|  | Are there any other instances or thoughts you would like to share on this topic? |
| **Additional** | Is there anything else you would like to add about your experiences or opinions on the integration of robots in healthcare settings? |
